# Calcium, Bioenergetics, and Parkinson’s Disease

**DOI:** 10.3390/cells9092045

**Published:** 2020-09-08

**Authors:** Enrico Zampese, D. James Surmeier

**Affiliations:** Department of Physiology, Feinberg School of Medicine, Northwestern University, Chicago, IL 60611, USA; enrico.zampese@northwestern.edu

**Keywords:** Parkinson’s disease, substantia nigra, dopaminergic neurons, Ca^2+^, mitochondria, bioenergetics, oxidative stress, neurodegeneration

## Abstract

Degeneration of substantia nigra (SN) dopaminergic (DAergic) neurons is responsible for the core motor deficits of Parkinson’s disease (PD). These neurons are autonomous pacemakers that have large cytosolic Ca^2+^ oscillations that have been linked to basal mitochondrial oxidant stress and turnover. This review explores the origin of Ca^2+^ oscillations and their role in the control of mitochondrial respiration, bioenergetics, and mitochondrial oxidant stress.

## 1. Introduction: The Duality of Intracellular Ca^2+^ Signaling

**Figure 1:**
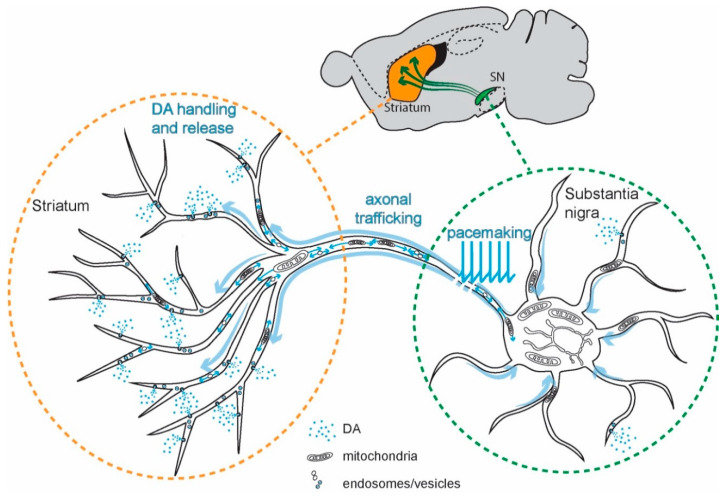
Schematic representation of a substantia nigra (SN) dopaminergic (DAergic) neuron, its axonal projections, and the factors contributing to its high bioenergetic demands. Inset: the positions of substantia nigra (SN, green) and the striatum (orange) and nigral projections to the striatum. Main: sources of high energy demand in SN DAergic neurons. In neurons, three main processes are responsible for the bioenergetic burden: the maintenance of the axonal tree through anterograde/retrograde transport, the propagation of action potentials (APs) followed by the re-establishment of ionic gradients, and the synaptic vesicles cycling. SN DAergic neurons have particularly high energetic demands because of the huge size of their axon, the autonomous pacemaking activity coupled to broad APs, and the high number of synapses or dopamine (DA) release sites. Note that DA release occurs not only from the axonal domain but also in the somatodendritic region.

The role of Ca^2+^ as a second messenger has been explored for decades [1,2,3,4]. One of the most intriguing features of Ca^2+^ that has emerged from this effort is its duality: Ca^2+^ signals are necessary for cellular health, but can also trigger dysfunction and death [5]. This duality also manifests itself in substantia nigra (SN) dopaminergic (DAergic) neurons (Figure 1). These neurons—whose degeneration is responsible for the core motor symptoms of Parkinson’s disease (PD) [6,7]—have large cytosolic oscillations in Ca^2+^ concentration ([Ca^2+^]). These oscillations play a key role in helping the neurons meet their bioenergetic needs, but they are also linked to cellular stress and vulnerability with aging and PD [8,9,10].

## 2. Neuronal Ca^2+^ Homeostasis

Spiking or synaptic activity can trigger transient elevations in cytosolic [Ca^2+^]. Generally speaking, there are three classes of plasma membrane (PM) proteins that underlie these transients. One class is formed by voltage-dependent Ca^2+^ permeable ion channels. These channels vary in their voltage-dependence, location, and kinetics, and are accordingly classified as L-type (Cav1.1–1.4), N-type (Cav2.1), P/Q-type (Cav2.2), R-type (Cav2.3), and T-type (Cav3.3) [11]. Voltage-dependent Ca^2+^ channels provide an elegant means of linking spiking and synaptic activity to intracellular machinery responsible for the control of other channels (e.g., Ca^2+^ activated K^+^ channels), transmitter release, metabolism, and gene expression [11]. Another class is formed by ionotropic receptors that are gated by neurotransmitters (e.g., nicotinic acetylcholine receptors) and flux Ca^2+^. The third class is formed by G_q_-linked G-protein coupled receptors (GPCRs) activated by neurotransmitters (e.g., metabotropic glutamate receptors) that do not flux Ca^2+^ themselves but generate ligands for receptors that release Ca^2+^ from intracellular stores [12,13]. Other channels that can participate in neuronal Ca^2+^ signaling include store-operated channels (SOCs) and transient receptor (TRP) channels [12]. The amplitude, kinetics, and spatial distribution of intracellular [Ca^2+^] transients triggered by these PM proteins are controlled by Ca^2+^ buffering proteins—proteins endowed with Ca^2+^ binding sites [14]; Ca^2+^ binding proteins also can serve as Ca^2+^ sensors and effectors by interacting with an extraordinary array of other signaling molecules [14].

Unlike most cations, the transmembrane gradient for Ca^2+^ between the extracellular space and the cytosol is typically several orders of magnitude. As a consequence, a sophisticated collection of molecular mechanisms exists to achieve this end [1,3,15,16]. While extracellular [Ca^2+^] is 1–2 mM, cytosolic [Ca^2+^] is generally maintained at nanomolar levels (approximately 100 nM) by pumps and exchangers that extrude Ca^2+^ across the plasma membrane (PM) or into intracellular stores. The PM is endowed with plasma membrane Ca^2+^ ATPases (PMCAs) and Na^+^/Ca^2+^ exchangers (NCXs) that expel Ca^2+^. PMCAs pump Ca^2+^ to the extracellular space by using adenosine triphosphate (ATP), while NCX takes advantage of the Na^+^ gradient created by the Na^+^/K^+^ ATPase to extrude Ca^2+^ to the extracellular space. Given their differences in affinity for Ca^2+^, it is likely that basal cytosolic [Ca^2+^] is largely governed by the PMCA and the NCX is engaged by activity that pushes local [Ca^2+^] higher, as during repetitive spiking [17].

The endoplasmic reticulum (ER) is the main intracellular Ca^2+^ store. Elevated ER [Ca^2+^] is maintained by the sarco/endoplasmic reticulum Ca^2+^ ATPases (SERCAs) that move Ca^2+^ from the cytosol. SERCAs serve to terminate cytosolic transients induced by PM processes and to counteract constitutive “leak” of Ca^2+^ from the ER itself. The ER is richly invested with Ca^2+^ buffer proteins (e.g., calreticulin) that differ from cytosolic buffers in their affinity and capacity to help stabilize the high (µM) luminal ER [Ca^2+^]. Ca^2+^ release from the ER is mediated by the inositol trisphosphate (IP_3_) receptor (IP_3_R) and the ryanodine receptor (RyR). IP_3_R is gated by IP_3_ generated by phospholipase C in response to the activation of GPCRs. The primary agonist of RyR is Ca^2+^ itself; thus, cytosolic Ca^2+^ transients can trigger Ca^2+^-induced Ca^2+^ release” (CICR) from the ER [12,13,18]. In this regard, it is important to remember that the ER is a morphologically complex system of cisternae and tubules spread through the neuron, extending into axons, dendrites, and spines [18,19,20]; thus, CICR creates a means of creating propagated Ca^2+^ waves from one region of a cell to another. In addition to the ER, other organelles—including mitochondria, the Golgi apparatus, lysosomes, and endosomes—act as Ca^2+^ stores and can contribute to shaping intracellular Ca^2+^ signaling events [15].

## 3. Ca^2+^ and Control of Mitochondria

Mitochondria are widely thought to be the “powerhouses” of neurons, meeting the bioenergetic demands of regenerative activity and neurotransmitter release. Hypothesized to be ancient bacterial symbionts, mitochondria have an outer membrane (OMM) perforated by relatively large pores. The OMM surrounds an inner membrane (IMM) with deep invaginations (cristae); in contrast to the OMM, the transit of molecules—and Ca^2+^—across the IMM is tightly regulated [21,22,23,24]. In healthy mitochondria, the electrochemical gradient across the IMM created by the electron transport chain (ETC) is very steep (~180 mV), providing a strong driving force for Ca^2+^ entry into the mitochondrial matrix [25,26]. Recently, a great deal of progress has been made in characterizing the molecular machinery responsible for regulating mitochondrial Ca^2+^ influx [26]. The influx of Ca^2+^ is controlled by the mitochondrial Ca^2+^ uniporter complex (MCUC). The MCUC is composed of the channel-forming unit, known as the mitochondrial Ca^2+^ uniporter (MCU) [27,28], and several accessory proteins that influence MCU gating [29,30]. These subunits limit MCU opening to periods when the intermembrane [Ca^2+^] is high (~10–20 µM). In physiological situations, this concentration is achieved inside neurons only in microdomains where diffusion is restricted [3,31]. Indeed, this kind of restricted diffusion space is created at specialized junctions between mitochondria and the ER [32,33,34,35,36,37], referred to as “mitochondria-associated membranes” (MAMs) [38,39,40]. There is also evidence that the MCUC is tailored to meet the needs of different subcellular compartments, like the nerve terminal [41].

Ca^2+^ is extruded from mitochondria by a Ca^2+^/H^+^ exchanger and—particularly in excitable cells—a Na^+^*/*Ca^2+^ exchanger (or Na^+^/Ca^2+^/Li^2+^ exchanger, NCLX) [25,42,43]. In contrast to Ca^2+^ entry through the MCU pore, the extrusion of Ca^2+^ by exchangers is relatively slow. This difference in dynamics shapes cytosolic Ca^2+^ signals [44,45,46,47]. Another possible mitochondrial exit pathway for Ca^2+^ is the mitochondria permeability transition pore (mPTP), which is generally thought to open only in pathological situations when matrix [Ca^2+^] gets too high [48]; however, the mPTP can also open transiently to modulate mitochondrial Ca^2+^ levels [26,49,50,51].

**Figure 2:**
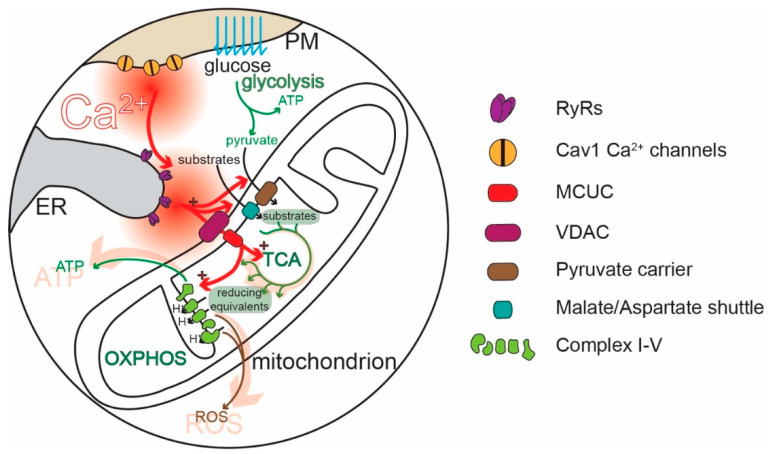
Effects of Ca^2+^ on mitochondrial bioenergetic metabolism and reactive oxygen species (ROS) production. The two primary sources of ATP in neurons are glycolysis and mitochondrial OXPHOS. During pacemaking, Ca^2+^ entry through Cav1 channels, coupled with CICR through RyRs, generates an elevation in [Ca^2+^]. Elevated [Ca^2+^] reaching the mitochondria favors the uptake of substrates by the mitochondria by acting on transporters and carriers on the IMM; Ca^2+^ taken up by mitochondria through the voltage-dependent anion channel (VDAC) and MCUC increases OXPHOS by enhancing the activity of the TCA cycle and by stimulating ATP synthesis by Complex V. The downside of this stimulation is an increase in the generation of ROS.

Although mitochondria regulate cellular functions in a variety of ways [52], one of their most important roles is the conversion of adenosine diphosphate (ADP) to ATP through oxidative phosphorylation (OXPHOS, Figure 2). OXPHOS complements glycolysis, generating 18 molecules of ATP for each pyruvate molecule produced from the metabolism of glucose [53,54]. Metabolic substrates, like pyruvate (also amino acids or ketones), are taken up by mitochondria and enter the tricarboxylic acid cycle (TCA), which converts them into reducing equivalents for the ETC. Complexes I-IV of the ETC located in the IMM use the reducing equivalents to transfer electrons to molecular oxygen and to pump protons (H^+^) from the mitochondrial matrix into the intermembrane space (IMS), between the IMM and the OMM. ATP synthase (complex V) then uses the H^+^ electrochemical gradient to convert ADP to ATP [50,55,56] (Figure 2). The rate of OXPHOS is modulated by cytosolic Ca^2+^ in several ways [57,58,59,60] (Figure 2). IMS Ca^2+^ stimulates the transport of metabolites into the matrix [59,61]. Ca^2+^ entry into the mitochondrial matrix through MCUC stimulates the generation of reducing equivalents by disinhibiting three key TCA dehydrogenases [62]. The mitochondrial matrix Ca^2+^ stimulates complex V [57]. In this way, Ca^2+^ signaling links regenerative activity to ATP production [63,64,65,66,67,68,69]. Ablating the MCU and preventing Ca^2+^ uptake in mitochondria leads to a compensatory upregulation of glycolysis, supporting the critical role of OXPHOS and its stimulation by Ca^2+^ for neuronal health [70].

## 4. SN DAergic Neurons and PD

The degeneration of SN DAergic neurons is responsible for the core motor symptoms—bradykinesia and rigidity—of PD [6,71,72]. These neurons are autonomous pacemakers: in the absence of external stimulation, SN DAergic neurons fire broad (~2–3 ms) action potentials (APs) at a regular frequency (1–4 Hz) [73,74,75,76]. SN DAergic neurons are part of the basal ganglia, and dopamine (DA) released from their axons modulates the activity of basal ganglia circuits controlling goal-directed actions and habits. The largest of the basal ganglia nuclei modulated by DA is the striatum. The autonomous pacemaking of SN DAergic neurons is modulated up and down by synaptic inputs [77], allowing bidirectional control of DA release, which in turn bidirectionally modulate basal ganglia circuits [78,79,80]. SN DAergic neurons also release DA from their somatodendritic membrane [81,82,83]. This release is known to modulate synaptic input to neighboring substantia nigra pars reticulata (SNr) GABAergic neurons that form a major portion of the basal ganglia interface with the rest of the brain [84]. The degeneration of SN DAergic neurons distorts cellular and network activity in the basal ganglia, resulting in the core motor symptoms of PD [6,71,72].

## 5. Ca^2+^ Signaling in SN DAergic Neurons

It’s our thesis that the vulnerability of SN DAergic neurons to aging and PD [85] is in large measure attributable to their distinctive phenotype [86]. This phenotype not only creates basal metabolic stress in the absence of overt pathology but also increases the impact of genetic mutations and environmental toxins linked to increased risk of developing PD. A key feature of this distinctive phenotype is the way Ca^2+^ signaling is engaged.

In all neurons, Ca^2+^ currents through voltage-dependent channels serve to promote and regulate regenerative spiking, as well as to link that activity to a variety of other processes. Specific channel subtypes play specific roles. For example, in presynaptic regions, Cav2 channels control exocytosis of neurotransmitters [11]. Ca^2+^ flux through somatodendritic Cav1.2 (L-type) channels control processes tied to spiking, as their open probability rises only when neurons spike. This feature allows them to generate Ca^2+^ signaling that is proportional to spike rate – an important variable not only for ion channels responsible for membrane excitability (e.g., K^+^ channels) but also the transcriptional machinery involved in processes like homeostatic plasticity [11].

Several types of ion channels—including Ca^2+^ channels—participate in the initiation and regulation of the autonomous rhythmic activity in SN DAergic neurons [73,87,88,89,90,91,92,93,94,95,96,97,98]. Most voltage-dependent Ca^2+^ channels (N-, P/Q-, R- and most L-type Ca^2+^ channels) require relatively depolarized potentials to activate, and they open only at membrane potentials above the spike threshold. For example, high voltage-activated, R-type (Cav2.3) channels contribute to somatic Ca^2+^ oscillations in SN DAergic neurons during spiking and help regulate spike patterning [99]. However, SN DAergic neurons express two types of voltage-dependent Ca^2+^ channels (Cav3 (T-type) and Cav1.3 (L-type)) that open at membrane potentials below the spike threshold and thus can help push the membrane potential to the threshold for spiking [100,101]. Indeed, Ca^2+^ imaging experiments have revealed cytosolic Ca^2+^ transients in SN DAergic neurons that begin well before the spike and then increase during it [101,102,103,104,105,106,107].

Cav3 channels (classified as low-voltage activated channels) activate at sub-threshold membrane potentials but inactivate with sustained depolarization. Although their activation and inactivation curves partially overlap, creating a “window current” that can destabilize membrane potential [108,109], their gating properties and sub-cellular location makes them particularly well-suited to the regulation of spiking patterns originating in the proximal somatodendritic region and axon initial segment [110]. In fact, recent quantitative Ca^2+^ imaging experiments have shown that the contribution of Cav3 channels to cytosolic Ca^2+^ transients is primarily in the proximal dendrites of SN neurons [104]. In this way, Ca^2+^ entry through Cav3 channels can increase the depolarization needed to trigger spikes (particularly in cases when the membrane is “released” from synaptic hyperpolarization that de-inactivates them [111]), but also help maintain the regularity of pacemaking and the duration of synaptically generated spike “bursts” by activating Ca^2+^-dependent SK K^+^ channels that pull the membrane potential in a negative direction [112].

Like Cav3 channels, Cav1 channels (L-type) containing the Cav1.3 pore-forming subunit [113,114,115] open at relatively hyperpolarized potentials and thus can contribute to the depolarization leading to the generation of rhythmic spontaneous spikes [116,117,118]. However, unlike Cav3 channels, Cav1 channels inactivate only modestly with depolarization, making them suitable for modulating more sustained changes in activity. Consistent with these properties, Cav1 channels drive a slow oscillatory potential (SOPs) in SN DAergic neurons when Na^+^ channels are blocked with tetrodotoxin [93,95,101,102,107,119,120]. However, the opening of Cav1 channels is not necessary for pacemaking, as asserted previously based upon experiments that employed dihydropyridines (DHPs) at concentrations where channel specificity is lost [89,96]. At nanomolar concentrations, where binding is specific to Cav1 channels, DHPs effectively inhibit cytoplasmic Ca^2+^ transients without changing the pacemaking rate [101]. Rather, the engagement of Cav1 channels increases the robustness of pacemaking that is largely driven by a cation leak channel (NALCN) [101,102,121,122]. Although SN DAergic neurons express both Cav1.2 and Cav1.3 channels, targeted genetic suppression of Cav1.3 channels mimics the effects of DHPs on dendritic Ca^2+^ transients, pointing to them as primary determinants of this feature of the phenotype [104]. Indeed, because of their gating properties, Cav1.3 channels are open through most of the pacemaking cycle [107].

During pacemaking, the cytosolic Ca^2+^ transient in the dendrites of SN DAergic neurons is estimated using quantitative Fura2 imaging to rise above 500 nM (near zones of Ca^2+^ entry or release, the concentration may reach into the microlar range). In part, the magnitude of this transient is attributable to low intrinsic Ca^2+^ buffering [123]. This allows Ca^2+^ to diffuse easily through the cytoplasm and control biochemical processes and gating of channels, like SK channels [124,125] and RyRs (triggering CICR) [126]. Indeed, Cav1.3 channels are strongly coupled to ER RYRs [127]. This coupling is responsible for much of the cytosolic Ca^2+^ transient during pacemaking (unpublished observations and [128]).

**Figure 3:**
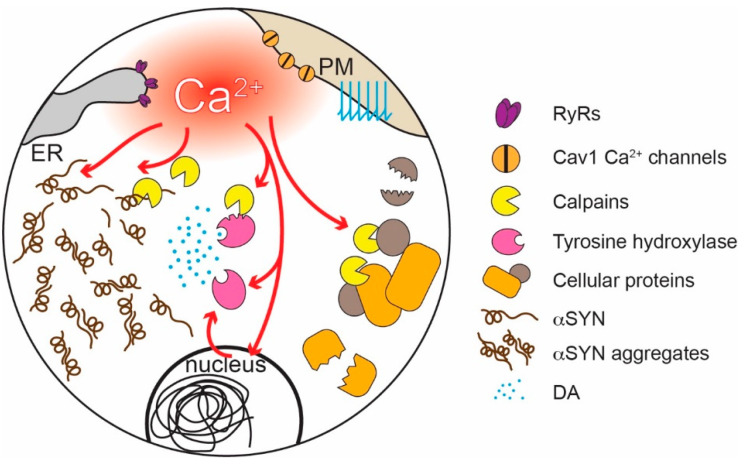
Toxic effects of elevated [Ca^2+^] in SN DAergic neurons. The engagement of Cav1 channels in pacemaking and the release of Ca^2+^ through RyRs generate elevated cytosolic Ca^2+^ levels that increase *α*-synuclein (*α*SYN) aggregation, directly or through the activation of calpains; Ca^2+^-activated calpains can also damage several intracellular proteins. Ca^2+^ and calpain cleavage can increase or dysregulate tyrosine hydroxylase activity and DA production, and DA oxidation can favor the aggregation of *α*SYN.

The purpose of Ca^2+^ signaling triggered by pacemaking in SN DAergic neurons is still being unraveled, but there are some clues. Ca^2+^ flux through Cav1 channels regulates the expression of genes coding for proteins responsible for the synthesis of DA, linking activity, and anabolic activity [129,130,131] (Figure 3). Another function of Cav1.3 channels in SN DAergic neurons is the control of mitochondrial OXPHOS (Figure 2). Unlike most neurons, SN DAergic neurons appear to have a high basal bioenergetic demand [132,133,134]. This demand may have its roots in several factors. The most important of these is likely to be the neuron’s massive axonal arbor [132,135]. This arbor creates an anabolic demand, as it has to be supplied with release-related proteins and lipids largely delivered by axonal transport from the somatic region [136,137,138,139]. The hundreds of thousands of DA release sites create an independent burden, as the release and recycling of synaptic vesicles is bioenergetically expensive [63,65,68,132]. As proteins and lipids within this arbor become damaged or dysfunctional, they have to be degraded, creating an additional catabolic demand [140]. Moreover, ionic gradients necessary to support regenerative activity throughout the axon poses a significant burden [133,141]. SN DAergic neurons also are constantly active, multiplying the demands associated with the axonal propagation of spikes (Figure 1) [8,133].

For those readers interested in the numbers, let us review them. In rodents, the axon of a single SN DAergic neuron can reach a length of over 40 cm and form more than 200,000 synapses, covering a significant portion of the striatum [132,142,143,144,145]. Neighboring ventral tegmental area (VTA) DAergic neurons also have relatively large axonal trees [146,147] but have far fewer transmitter release sites (~12,000–30,000 [132]). Although an order of magnitude less than SN DAergic neurons, this is still substantially greater than many other neurons [148,149]. In the human brain, SN DAergic neurons may have an order of magnitude greater number of release sites than those in the mouse, possibly because of forebrain evolution [132,134,150].

Direct evidence of the bioenergetic burden posed by the axon of SN DAergic neurons comes from a novel in vitro study [151]. The authors not only confirmed that SN DAergic neurons have longer and more branched axons than VTA DAergic neurons, but also that axonal size was directly correlated with oxygen consumption rate (OCR, an index of mitochondria OXPHOS) and mitochondrial oxidant stress. Reducing the size of the axonal arbor decreased OCR, oxidant stress, and vulnerability to parkinsonian toxins. Interestingly, inhibiting Cav1 Ca^2+^ channels decreased OCR [151]. In a follow-up study in vivo, the authors demonstrated that increasing axonal size in SN DAergic neurons increased their vulnerability to mitochondrial toxins [152]—solidifying the connection between axonal arbor size and mitochondrial stress.

If we accept the proposition that SN DAergic neurons have a high basal bioenergetic demand, how do they satisfy that demand? As noted above, OXPHOS is an efficient means of generating ATP from glucose, fatty acids, and amino acids. It has long been thought that ATP levels were maintained by ATP-mediated feedback control of complex V [153,154,155,156]. The problem with this kind of control mechanism is speed. SN DAergic neurons dynamically regulate basal ganglia circuits controlling escape, attack, and habitual behaviors. If ATP levels fall and DAergic neurons slow their spiking or release of DA because of flagging ATP levels [157,158], the organism’s movement will begin to slow, making it vulnerable. Thus, there must have been strong evolutionary pressure to develop a control strategy that does not depend upon feedback. In muscle, a feedforward, “anticipatory” control mechanism is used to drive OXPHOS [159,160]. A similar mechanism is in place in SN DAergic neurons where the Cav1.3 channel triggered ER release of Ca^2+^ through RYRs “injects” Ca^2+^ into mitochondria at MAMs—stimulating OXPHOS in anticipation of need. Indeed, inhibition of mitochondrial OXPHOS or glucose deprivation causes them to hyperpolarize and stop spiking [157,158,161,162]. Interestingly, a recent paper has confirmed in hippocampal neurons that Ca^2+^ influx through Cav1 channels combined with CICR can regulate mitochondria ATP production, although in these non-pacemaking neurons mitochondrial contribution to cell bioenergetic seems to be relatively small and this mechanism is activated only upon stimulation [163]. At axonal DA release sites, Ca^2+^ stimulated mitochondrial OXPHOS is complemented by another feedforward system in which DA transiting the cytosol is metabolized by monoamine oxidase (MAO) anchored to the outer membrane of mitochondria; in so doing, MAO generates an electron that is shuttled to the ETC, which supports the electrochemical gradient used by complex V to generate ATP [164]. Thus, feedforward control of mitochondrial OXPHOS is a mechanism by which SN DAergic neurons can maximize their functionality and promote organismal survival.

## 6. Why are SN DAergic Neurons Preferentially Vulnerable in PD?

Several theories have been advanced to explain the selective vulnerability of SN DAergic neurons in PD. The oldest is that DA is responsible. DA is a reactive molecule that when oxidized or metabolized can damage a variety of cellular proteins and lipids, most importantly *α*-synuclein (*α*SYN) [165,166,167,168,169,170,171,172,173,174] (Figure 3). In human mesencephalic DA neurons, cytosolic DA may be particularly high [175], allowing Cav1 channel-driven mitochondrial oxidant stress to significantly increase DA oxidation; the combination of mitochondrially generated reactive oxygen species (ROS) that escape into the cytoplasm and oxidized DA promotes not only misfolding of *α*SYN but also damage to lysosomal proteins that play a role in clearing misfolded *α*SYN [175]. Recent work also has shown that monoamine oxidase (MAO) metabolism of cytosolic DA in axons and distal dendrites increases mitochondrial oxidant stress by shuttling electrons to the ETC, creating a novel interaction between DA and mitochondria that could have pathological consequences [164]. That said, while DA might accelerate pathogenesis in PD, it is not the sole culprit. It has become increasingly clear that other transmitter phenotypes, particularly cholinergic and adrenergic neurons, also are highly vulnerable in PD [8,72].

While transmitter phenotype may not be a universally shared trait of vulnerable neurons, other traits are shared [8,72,135]. One cluster of shared traits is modest cytosolic Ca^2+^ buffering, a slow, broad-spike, autonomous pacemaking, and Cav1.3 channel opening that triggers CICR; together, these traits result in the generation of large Ca^2+^ transients several times a second. In contrast, VTA DAergic neurons are pacemakers, but do not manifest large [Ca^2+^] transients and are largely spared in PD [97,105,122,176]. This difference in Ca^2+^ handling is attributable in part to higher expression of the Ca^2+^ buffering protein calbindin-D28k (CB-D28k) [90,177,178,179]. CB-D28k expression levels between SN and VTA and within SN itself are correlated with vulnerability in experimental models and in clinical PD [180,181,182,183,184,185,186]. Interestingly, intracellular Ca^2+^ chelation or over-expression of CB-D28K can protect DAergic neurons against the deleterious effects of Ca^2+^ entry, including a gain of function mutation in the TRP channel Trp-4 [187].

How might physiological levels of Ca^2+^ experienced by at-risk neurons promote PD pathology? There is growing evidence that Ca^2+^, directly and indirectly, promotes *α*SYN pathology—a hallmark of many forms of PD [72,188,189,190]. The negatively charged C-terminal region of *α*SYN inhibits misfolding and aggregation [191]. Binding of Ca^2+^ to this region attenuates electrostatic repulsion and promotes the formation of oligomers and higher molecular weight aggregates both in reconstituted preparations and in cells [192,193,194,195,196]. Ca^2+^ also promotes *α*SYN aggregation by enhancing calmodulin and membrane binding [197,198]. Conversely, increasing Ca^2+^ buffering decreases *α*SYN aggregation [199]. High (low micromolar) cytosolic [Ca^2+^]—like that achieved in SN DAergic neuron dendrites—also activates proteases known as calpains, which cleave a variety of cellular proteins [200], including *α*SYN and tyrosine hydroxylase, a key synthetic enzyme for DA (Figure 3) [201,202,203]. Calpain cleaves the C-terminal region of *α*SYN discussed above, promoting its aggregation [204]. Pharmacological or genetic inhibition of calpains reduces *α*SYN cleavage, aggregation, and toxicity [205] and is neuroprotective in PD models [206].

Interestingly, Ca^2+^ signaling also can be shaped by *α*SYN aggregates. At high concentrations, *α*SYN can induce the formation of Ca^2+^ permeable pores in membranes and enhance the activity of SERCA, possibly contributing to Ca^2+^-induced *α*SYN aggregation and damage [207,208,209,210,211,212]. Elevated cytosolic [Ca^2+^] also might promote the spreading of *α*SYN pathology, as cytosolic Ca^2+^ enhances *α*SYN release [213,214]. Moreover, another way in which *α*SYN affects Ca^2+^ homeostasis is by modulating ER-mitochondria Ca^2+^ transfer at the MAMs [215].

Another way in which Ca^2+^ signaling may increase neuronal vulnerability is through enhancing the production of superoxide and damaging ROS by mitochondria (Figure 2). The movement of electrons along the mitochondrial ETC is inevitably associated with electrons “jumping” to molecular oxygen and the generation of superoxide and ROS, primarily by mitochondrial complex I and III [50,55,216,217,218]. ROS can damage deoxyribonucleic acid (DNA), lipids, and proteins [218]. Although mitochondria are endowed with a variety of antioxidant defenses [219], these systems are imperfect [217,219]. Feedforward ETC stimulation not only results in longer respiratory bouts but also periods of stimulation during which there is little ATP demand and high mitochondrial membrane potential, a situation that is particularly likely to result in superoxide/ROS production [10,220]. Indeed, SN DAergic neurons have high levels of mitochondrial oxidant stress “at rest” and in the absence of pathology, as shown in primary neuronal cultures [106,209] and ex-vivo slices from mice [104,105]. By contrast, mitochondrial oxidant stress in VTA DAergic neurons is much lower [105,106,209]. Suppressing feedforward mitochondrial stimulation by inhibiting Cav1 Ca^2+^ channels lowers mitochondrial oxidant stress [104,105,106], supporting the connection between normal Ca^2+^ signaling and oxidant stress.

Mitochondrial ROS can damage mitochondrial proteins and DNA (mtDNA). The accumulation of mtDNA deletions characteristic of sustained oxidant stress is a well-described feature of SN DAergic neurons in aged humans and PD patients, in contrast to other types of neuron [221,222,223,224,225,226]. It is important to mention that mtDNA encodes only 13 proteins, all critical components of the OXPHOS machinery; thus, once a cell accumulates enough mtDNA deletions, the ability of mitochondria to produce ATP will be compromised [24,227]. In addition, because they are proximal to the sites of ROS generation, ETC proteins are prone to damage. Loss of complex I, which is the largest of the ETC complexes and a major source of ROS, is a key feature of SN DAergic neurons in PD patients [228,229,230,231,232].

Interestingly, in the somatodendritic regions of SN DAergic neurons, mitochondrial mass is paradoxically low [233]. Recent work has confirmed this observation and explained why it is this way. It turns out that the high mitochondrial oxidant stress driven by Cav1 Ca^2+^ channel-dependent stimulation of OXPHOS results in mitochondrial damage and elevated rates of mitophagy in SN DAergic neurons [104]. Systemic administration of DHPs to mice at concentrations that inhibit Cav1 channels decreases mitochondrial oxidant stress, slows mitophagy, and normalizes mitochondrial mass in SN DAergic neurons over the course of about a week [104]. Although it remains to be determined whether macroautophagy or mitochondrial-derived vesicles (MDVs) turnover is engaged by this process [234,235], this challenge is likely to compromise the ability of neurons to deal with other protein degradation tasks, like clearing *α*SYN aggregates. Moreover, with age the efficiency of macroautophagy declines [236]. This could have particularly dire consequences for SN DAergic neurons as their autophagic capacity may be pushed close to its limit by the combined catabolic demands associated with mitochondria and *α*SYN aggregation created by the massive axonal arbor. Aging, and any other stressor, like a genetic mutation compromising mitochondrial or autophagic function or an environmental toxin that exacerbates mitochondrial stress, could create a tipping point for degeneration. Interestingly, several studies of non-neuronal cells obtained from sporadic or familial PD patients have revealed bioenergetic and mitochondrial deficits [237,238,239,240,241,242,243,244,245,246], suggesting that there may be a systemic impairment in metabolism in PD, but only in neurons with little spare metabolic capacity (e.g., SN DAergic neurons) does this defect result in degeneration.

## 7. Is Mitochondrial Dysfunction Sufficient to Cause PD?

Although there are clear signs of mitochondrial pathology in the SN of PD patients, there is a continuing debate about whether mitochondrial dysfunction is a root cause of PD or whether it is merely a tombstone or consequence of pathology. For some time, there was little debate. At high enough doses, mitochondrial toxins, like rotenone and 1-methyl-4-phenyl-1,2,3,6-tetrahydropyridine (MPTP), effectively kill SN DAergic neurons in mice and primates and induce a parkinsonian like state within a matter of hours [247,248]. However, drugs that effectively blunt the toxicity of these compounds have consistently failed in human clinical trials [249,250,251]. An influential paper on this topic demonstrated that impairing complex I function in DAergic neurons by deleting one of its subunits (Ndufs4) had little effect on them and did not alter the sensitivity to toxins like rotenone [252,253,254,255]. That said, making complex I insensitive to rotenone or MPTP by knocking down p13 confers neuroprotection in toxin models of PD [256].

More recent attempts to determine the possible impact of mitochondrial dysfunction on PD pathogenesis have turned to genetic strategies. As already mentioned above, it is widely assumed that neurons need mitochondria, particularly in axons [137,257]. Reduced expression of molecular motors associated with axonal transport has been observed in tissue from early-stage PD patients [258]. Exposure to PD toxins (6-Hydroxydopamine, 6-OHDA, or MPTP metabolites) decreased anterograde mitochondrial axonal transport in primary cultures of rodent DAergic neurons [259,260] and in transgenic zebrafish [261].

More compelling evidence of the importance of mitochondrial dynamics in SN DAergic neurons comes from studies based on the manipulation of the molecular machinery responsible for these dynamics. Mitochondria undergo fusion and fission, allowing them to exchange mtDNA and other components (fusion) and to generate smaller isolated organelles (fission) that can be easily transported through the cell or destined for degradation [262,263]. Diminishing mitochondrial fission upon deletion of Drp1 leads to the depletion of mitochondria from the axons of SN DAergic neurons, progressive loss of SN striatal projections, and neuronal loss in SN [264]. Similarly, the deletion of mitofusin 2 (but not mitofusin 1), which is involved in mitochondrial fusion and ER-mitochondria tethering (see below), causes decreased mitochondrial transport and axonal degeneration in SN DAergic neurons [265,266].

Another strategy to test the role mitochondria in SN DAergic neurons is to target mtDNA. The “MitoPark” mouse is based on a DAergic-specific deletion of the mitochondria transcription factor Tfam, compromising mitochondrial transcription and mtDNA maintenance, disrupting the synthesis of critical subunits of the OXPHOS machinery [267]. Within weeks of birth, DAergic neurons in MitoPark mice have dysmorphic mitochondria, impaired spiking; later, SN DAergic neurons degenerate and mice manifest a parkinsonian phenotype [267,268]. Similarly, targeting the endonuclease (PstI) to mitochondria in DAergic neurons, which causes mtDNA damage and OXPHOS dysfunction, results in a slow loss of SN DAergic neurons and motor impairments [269]. These studies demonstrate that mitochondria are necessary for normal functioning and survival of SN DAergic neurons, but they do not resolve the issue about whether the loss of complex I function seen in the SN of PD patients is a driver of pathogenesis.

In an attempt to directly target complex I, a subunit of complex I (Ndufs4) was deleted in DAergic neurons of mice. However, these mice don’t manifest a parkinsonian phenotype [253,254,255,270]. These results need to be interpreted with caution however as Ndufs4 deletion only partially decreases in complex I activity [270,271]. A more complete disruption of complex I activity, like that achieved by deletion of the catalytic subunit (Ndufs2), would be more informative.

The most compelling evidence for the involvement of mitochondria in PD pathogenesis is based upon an examination of the consequences of genetic mutations associated with relatively rare familial forms of the disease [272,273,274]. Many of these mutations modulate mitochondrial homeostasis [275,276,277,278,279,280,281,282,283], dynamics [284,285,286,287,288,289], redox status [105,290], and biogenesis [291,292].

Particularly intriguing is the role of parkin (PARK-2) and PTEN-induced kinase 1 (PINK1, PARK-6) in mitochondria quality control, especially because in SN DAergic neurons mitochondria have elevated oxidant stress, mtDNA damage, and turnover rates (see above). PINK1 (PARK-6) and parkin (PARK-2) cooperate in a pathway that tags damaged mitochondria for mitophagic degradation [293]. Briefly, PINK1 (PARK-6) is constitutively imported and degraded in healthy mitochondria, but upon mitochondrial damage, it accumulates on the OMM, where it recruits and activates parkin (PARK-2) [294]. Parkin (PARK-2), in turn, ubiquitinates OMM proteins and induces the formation of the autophagosome that will engulf the damaged mitochondria, leading to mitophagy [293,295,296]. Another way in which PINK1 (PARK-6) and Parkin (PARK-2) ensure mitochondrial quality control is through the generation of MDVs that contain damaged mitochondrial components targeted for lysosomal degradation [235]. Loss of function mutations in PINK1 (PARK-6) and parkin (PARK-2) mutations observed in familial PD patients suggest that a defect in the elimination (and the consequent accumulation) of dysfunctional mitochondria can increase the already elevated mitochondrial stress of SN DAergic neurons.

These familial mutations can also affect mitochondrial Ca^2+^ signaling. Deletion of PINK1 (PARK-6) is associated both with either decreased mitochondria Ca^2+^ uptake due to depolarization [297] or impaired mitochondrial Ca^2+^ efflux, which facilitates mitochondrial Ca^2+^ overload [298,299,300]; parkin (PARK-2) regulates the levels and the turnover of the MCUC regulators MICU1/2 [301]. In both cases, the disruption caused by deletion or loss of function mutations in these genes could be attributed to poor quality control. PD-linked mutations in leucine-rich repeat kinase 2 (LRRK2, PARK-8) increase the expression of MCU and MICU1 [302] and decrease mitochondrial Ca^2+^ efflux [303]. In zebrafish, inhibition of mitochondrial Ca^2+^ influx protects neurons against the effects of mutations mimicking the functional effects of those seen in PD patients [304,305], just as does inhibition of Cav1 Ca^2+^ channels responsible for mitochondrial Ca^2+^ influx in rodent SN DAergic neurons [102,105,306,307,308,309,310].

Another key site that is modulated by genetic mutations associated with PD is the MAM [215,291,311,312,313,314,315,316,317,318,319]. Dysregulation of MAMs has emerged as a key feature of neurodegenerative processes and PD in particular [318,320,321,322,323]. Many of the proteins encoded by genes mutated in familial PD regulate ER-mitochondria junctions, including *α*SYN (PARK-1/4) [215,311,316,317], Parkin (PARK-2) and PINK1 (PARK-6) [291,312,313,319,324], DJ-1(PARK-7) [314,315,319] and LRRK2 (PARK-8) [313,325]. Given the key role played by mitochondria in SN DAergic neurons, any dysregulation in the Ca^2+^ signals to the mitochondria could either impair the feed-forward mechanism that maintains the supply of ATP or exacerbate the already high oxidant burden experienced by the organelles.

One particularly bothersome aspect of this literature is that mice with PD-linked mutations do not develop a true parkinsonian phenotype. This is true for both the recessive mutations that are tightly linked to mitochondria (PARK-2, 6, 7) [326,327,328] and for the dominant mutations with more complex linkages to mitochondria [327,328]. Why this is the case is unclear, but, likely, human aging (the biggest risk factor for PD) is not faithfully captured in rodents.

## 8. Other Vulnerable Neuronal Populations

If Ca^2+^ and feedforward control of mitochondrial OXPHOS are the keys to the vulnerability of SN DAergic neurons in PD, other neuronal populations at-risk in PD should have a similar phenotype. Many other neurons, particularly in the brainstem, are vulnerable in PD [8,72,135]. In the Braak staging model, the earliest signs of Lewy pathology (LP) are in the dorsal motor nucleus of the vagus (DMV) [190,329,330]. As discussed elsewhere, the relationship between LP and neurodegeneration and death is far from clear [72,331]. In the SN, LP trails neurodegeneration [72,331]. Nevertheless, this line of study underscores the fact that several types of neurons distributed along the neuroaxis are vulnerable in PD, warranting a comparative analysis. In general, these other populations have not received the same level of attention as SN DAergic neurons. However, some intriguing similarities have already begun to emerge.

The cholinergic neurons of DMV are among the first neurons affected by LP, according to the Braak staging [330]. They form very long and branched axons that reach many gastro-intestinal-related organs, from the esophagus to the colon [332]. As with SN DA neurons, their firing activity has been described as a slow pacemaker, engaging various Ca^2+^ channels, including Cav1.2, Cav1.3, and Cav2 [333,334,335,336]. More importantly, DMV neurons manifest cytosolic Ca^2+^ oscillations and elevated mitochondrial oxidant stress (resembling SN DAergic neurons) [336,337,338]. Another vulnerable population of cholinergic neurons are in the pedunculopontine nucleus (PPN). PPN neurons are heterogeneous, being comprised of glutamatergic, cholinergic, and GABAergic neurons [339,340,341]. Cholinergic neurons are the most vulnerable [342,343,344]. Like SN DAergic and DMV cholinergic neurons, PPN cholinergic neurons are autonomous pacemakers with robust cytosolic Ca^2+^ oscillations (unpublished observations) and long, highly branched axons [345,346,347,348,349,350,351,352].

Two other PD vulnerable cell types have been studied in some depth. Adrenergic neurons in the locus coeruleus (LC) are among the first to degenerate in PD [190,353,354]. LC neurons show spontaneous rhythmic firing, whose frequency correlates with waking or sleeping states and sensory stimulation [355,356,357,358,359,360,361,362]. As in SN DA neurons, LC neurons engage L-type and T-type Ca^2+^ channels in pacemaking [128,363,364] and have low intrinsic cytosolic Ca^2+^ buffering and high levels of mitochondrial oxidant stress [128]. In addition, as other vulnerable neurons studied, LC neurons have long, highly branched axonal arbors [365,366,367,368]. Another vulnerable neuronal population resides in the raphe nuclei (RN). Again, these neurons have highly branched axonal arbors [369,370,371,372,373,374]. RN neurons are active during the waking state but slow down during sleep [375,376,377,378,379,380,381,382]. Spiking of RN neurons is sensitive to inhibition of Cav1 Ca^2+^ channels [383], but precisely why this is the case is unclear.

Thus, the available data indicates that an extensive axonal branching, autonomous pacemaking, and Cav1 channel-mediated feedforward control of mitochondrial OXPHOS (and the consequent mitochondrial oxidant stress) might be key features determining neuronal vulnerability in PD [8,72,132]. Instead, the neurotransmitter phenotype per se does not seem to represent an intrinsic risk factor in PD: not all DAergic, serotoninergic, adrenergic, and cholinergic neurons are vulnerable in the disease. However, it is noteworthy that the vulnerable neurons exert a widespread neuromodulatory role rather than releasing conventional fast neurotransmitters (i.e., glutamate and GABA) [72].

## 9. Conclusions and Future Directions

Ca^2+^ signaling plays a central role in many aspects of neuronal function. One under-appreciated role is in the control of neuronal bioenergetics. In SN DAergic neurons, Ca^2+^ entry through Cav1 Ca^2+^ channels couples activity to feedforward control of mitochondrial OXPHOS. This coupling has two apparently unintended consequences. One is a robust oscillation in cytosolic [Ca^2+^]; another is the excessive production of ROS by mitochondria. Both unintended consequences can have deleterious consequences over time (Figure 2 and Figure 3). This situation may be an example of antagonist pleiotropy [384,385]. Pacemaking-dependent Ca^2+^-mediated feed-forward stimulation of mitochondria should confer an advantage in the early stages of life when an animal (reprising the example used above) needs to escape predators or hunt for food and ultimately survive to mate. Only later in life, past reproductive age, this design may have negative consequences [141,386]. The average age of diagnosis with PD is about 60 years old [387]. As a consequence, it has only been relatively recently with the extension of the average lifespan that the incidence of PD has risen [388,389,390].

A fundamental question is then whether alleviating mitochondria oxidant stress could safely prevent or alleviate the progression of PD. Many of the early attempts at disease modification in PD have targeted mitochondrial ROS signaling, but all of these have failed to show efficacy [249,250,251]. Recently, epidemiological studies identified a correlation between PD risk and the use of DHPs Cav1 channel inhibitors [391,392,393,394,395], and preclinical studies supported this connection [102,306,307,308,309,310,396,397]. However, a Phase III clinical trial with the DHP isradipine did not show any benefit of the drug versus the placedo in slowing the progression of PD [398]. This trial may have failed for many reasons, but there are two obvious possibilities. One is that even in early-stage PD patients there has been a substantial loss of DAergic neurons and the processes driving the disease forward have changed to ones (e.g., inflammation) that will not be responsive to Cav1 channel inhibition. The epidemiological data supporting a protective role for DHPs invariably comes from presymptomatic patients that may be 5–10 years away from the typical age of PD diagnosis (~60 years of age). Unfortunately, the development of predictive biomarkers of disease onset and progression remains one of the main challenges facing the PD field [399,400]. The other (and to our mind more likely possibility) is that there was inadequate target engagement (Cav1 channel inhibition) with the twice daily, 5 mg immediate-release format isradipine pill that was used in the STEADY-PD III trial. After oral delivery, DHPs like isradipine are cleared within hours and pharmacokinetic modeling suggests that for most of the day plasma (and brain) isradipine concentrations were well below the threshold for protection determined in preclinical studies [306]. In retrospect, the use of a controlled release format of the drug that would have produced a sustained elevation in plasma (and brain) drug concentration, mimicking the preclinical studies, could have resulted in a different outcome.

As outlined above, there may be other Ca^2+^ channels that could be targeted in PD. For example, DAergic neurons derived from induced pluripotent stem cells from familial PD patients are protected from rotenone toxicity upon Cav3 channel inhibition [401], while knock-out of Cav2.3 channels protects mice from MPTP neurotoxicity [99]. Negative modulators of the MCUC [402] could decrease mitochondrial oxidant stress. Agonists of lysosomal Ca^2+^ channels could enhance lysosomal exocytosis and diminish αSYN accumulation [403]. However, all of these targets come with caveats given that these channels are widely distributed in the body and brain; as a consequence, it may be difficult to achieve enough biological effect with any one drug to alter disease course without causing intolerable side-effects. In this situation, intersectional approaches may prove worthwhile. That is, to target a combination of proteins in vulnerable neurons to achieve specificity of action, without bringing about unacceptable side-effects.
